# Characterization of HER2 Expression Levels, Including HER2-Ultralow, in a Retrospective Male Breast Cancer Cohort

**DOI:** 10.3390/life16060947

**Published:** 2026-06-03

**Authors:** Maximilian Marhold, Alexa Binder, Zsuzsanna Bago-Horvath, Stefan Konrad, Daniela Kauer-Dorner, Rupert Bartsch, Ruth Exner, Kerstin Wimmer

**Affiliations:** 1 Division of Oncology, Department for Medicine I, Medical University of Vienna, Währinger Gürtel 18-20, 1090 Vienna, Austria; maximilian.marhold@meduniwien.ac.at; 2 Institute for Pathology, Medical University of Vienna, 1090 Vienna, Austria; 3 Department of Radiooncology, Medical University of Vienna, 1090 Vienna, Austria; stefan.konrad@meduniwien.ac.at (S.K.); daniela.kauer-dorner@meduniwien.ac.at (D.K.-D.); 4 Department of General Surgery, Medical University of Vienna, 1090 Vienna, Austriakerstin.wimmer@meduniwien.ac.at (K.W.)

**Keywords:** male breast cancer, breast cancer, HER2, HER2-ultralow, HER2-low

## Abstract

Purpose: Male breast cancer (maleBC) is an orphan disease. Aside from age, risk factors include genetic mutations and conditions like Klinefelter syndrome or other reasons of hypogonadism. Treatment is based on standards in women, but biological differences may exist, with maleBC exhibiting higher rates of hormone-receptor expression. Limiting data exists regarding the rate of low/ultralow HER2 expression, a predictive biomarker for the antibody–drug conjugate (ADC) trastuzumab deruxtecan (T-DXd) in HER2-negative disease in women. Materials and Methods: We conducted a retrospective single-center analysis of clinicopathological features of maleBC at a tertiary cancer center. We identified a cohort of 57 maleBC patients, described demographic, pathological, prognostic and surgical and systemic treatment data and evaluated frequencies of low and ultralow HER2 expression. Results: The mean age was 64.3 (SE ± 1.79) years; 94.7% (*n* = 54) of patients presented with early/nonmetastatic breast cancer and 40.7% (22 of 54) of patients exhibited nodal involvement. Most patients (92.6%, 50 of 54 patients) had luminal disease and approximately one third of patients received chemotherapy. Endocrine therapy was administered in 87.7% (*n* = 50/57) of cases. For 52 of the patients included, full receptor status data including HER2 IHC scoring and/or histological specimens were available. IHC slides of tumor specimens from 24 patients with either historically reported “HER2 negative” or “HER2 0” expression were available for reassessment; 79.2% (*n* = 19 of 24) of these tumors exhibited either HER2-low or -ultralow expression. In the whole cohort, rates of HER2-low and -ultralow expression were 75.0% (39 of 52) and 5.8% (3 of 52 patients), respectively. The median follow-up was 7 years. Six deaths occurred in the total population (*n* = 57). The median event-free survival (EFS) was 8.39 years (95% CI 7.36–11.35). No statistically significant associations were observed between HER2 expression categories and clinicopathological variables including grade, ER status, nodal status, genetic variant status, age, Ki67 index, or overall survival events. Conclusions: In this cohort of maleBC patients, high rates of combined HER2-low and -ultralow expression were observed upon reassessment of tumors with historically negative HER2 status, shedding light on potential ADC eligibility in male breast cancer.

## 1. Introduction

Breast cancer (BC) is a predominantly female malignancy. However, it also affects men, albeit at a significantly lower incidence. According to the American Cancer Society, maleBC accounts for approximately 1% of all BC cases, with an estimated 2620 new diagnoses in the United States in 2023 [1]. Despite its rarity, the implications for affected individuals are substantial, as maleBC often presents at a later stage due to lack of awareness and misconceptions regarding the disease and thus may confer a worse prognosis compared to female BC [2] while stage-adapted outcomes are comparable [3].

The etiology of maleBC is multifactorial, involving genetic, hormonal, and environmental factors. Genetic predispositions, particularly mutations in the *BRCA2* gene, have been identified as significant risk factors [2].

Histologically, the vast majority of maleBC cases are luminal tumors of no special type (NST) [4] with a higher rate of estrogen- and androgen-receptor positivity reported. In contrast, lobular BC is rare in men [5]. Besides *BRCA1/2* mutations, other molecular differences have been described for maleBC when compared to female BC, e.g., expression levels of cell-cycle regulators p27Kip1 and p21Waf1 [6], as well as a higher rate of somatic mutations in genes linked to DNA repair [7]. However, the depth and size of molecular analyses performed for maleBC remain limited. Until recently, sequencing data of patient populations were restricted to hereditary forms of maleBC only [8,9] and genetic data of unselected cohorts of maleBC were limited to small case series, due to its orphan disease status.

Treatment modalities for maleBC are analogous to those employed in female BC [10]. Given the high rate of ER-positive tumors, endocrine therapy with tamoxifen remains the backbone of systemic treatment as aromatase inhibitors result in incomplete estradiol suppression in men [11]. However, due to the limited number of male patients, research on treatment outcomes specific to this population remains scarce [4]. While additional GnRH analogs are recommended in the case of aromatase inhibitor therapy [12], their role in conjunction with tamoxifen remains ill-defined.

Recently, low expression of HER2 (defined as HER2 1+/2+ by immunohistochemistry in the absence of *ERBB2* gene amplification) has gained significant attention due to the results of the DESTINY-Breast04 trial, showing the superiority of the HER2-targeting antibody–drug conjugate (ADC) trastuzumab deruxtecan (T-DXd) over chemotherapy of physicians’ choice in previously treated, metastatic, HER2-low-expressing BC [13]. Superiority of T-DXd over conventional chemotherapy was also demonstrated in the population of patients with ultralow HER2 expression [14] in the DESTINY-Breast06 trial. Conflicting rates of low HER2 expression in maleBC ranging from 27% to 76% were reported in three recent publications [15,16,17], with the highest rate in the largest cohort suggesting a potentially higher rate of low HER2 expression in male compared with female BC [15]. In addition, the frequency of ultralow HER2 expression specifically in male breast cancer is largely unclear, with Nobbe et al. reporting it in 47 of 120 male breast tumors (39%) in a German cohort [16] and Shang et al. reporting 13 HER2-ultralow tumors within a Chinese cohort of 106 patients (12%) [18].

This retrospective chart review was initiated to further elucidate the frequency of low and ultralow HER2 expression and its potential effects on outcome in maleBC patients.

## 2. Materials and Methods

### 2.1. Retrospective Patient Collection and Descriptive Analyses

Retrospective methodology using long observation time was chosen for this analysis in order to yield high patient case numbers of this orphan disease. In total, 57 male patients, 54 with early disease and 3 with metastatic disease, diagnosed with BC between January 2003 and October 2024 were identified using the in-house patient administration system (AKIM, SAP SE, Walldorf, Germany) and chemotherapy order software (CATO^®^, V2.50.9, Becton Dickinson Austria GmbH, Vienna, Austria). Information with regard to tumor characteristics, treatment and outcome was obtained by retrospective chart review. Missing categorical clinicopathological variables were coded as ‘unknown’ and included as separate categories in descriptive analyses. For survival analyses, patients without documented events were censored at the date of last follow-up. Surgical complications were assessed according to the Clavien-Dindo classification [19]. Data concerning tumor biology was derived from the last available histologic report. This study was conducted in accordance with the ethical regulations of our institution (committee vote 2244/2025) and the principles of the Helsinki Declaration (2013) were followed.

### 2.2. ER/PgR and HER2-Analyses

IHC slides of patients with HER2 IHC scores of “0” or stated as “negative” in their records were reassessed by two experienced breast pathologists of our institution for expression of HER2 by counting the value of positive cells as a percent and by considering novel diagnostic standards including recommendations for HER2-low and -ultralow (incomplete and faint membrane staining in ≤10% of tumor cells) scoring, including ASCO/CAP criteria [20]. Blinded analysis was not possible as the same pathologists performed initial and historic evaluation. For five patients who had surgery or biopsy in external institutions or hospitals, IHC slides and/or histological information could not be obtained.

### 2.3. Statistical Considerations

For the calculation of EFS, defined as the time to recurrence or death in patients with early-stage disease and the time to first progression or death in patients with metastatic disease, the Kaplan–Meier method was used and the number and proportion of survival events, with their median survival time and survival rates and their corresponding 95% CIs, were reported. Statistical analyses were performed using Microsoft Excel and IBM SPSS Statistics Version 32.0. Kaplan–Meier curves were calculated using SPSS. Precise 3- and 5-year EFS values were calculated using Statistics Kingdom^®^ (https://www.statskingdom.com/, date of request 13 December 2024). Associations between HER2 expression categories (negative, ultralow, low, positive) and categorical clinicopathological variables were analyzed using the Fisher–Freeman–Halton exact test. Continuous variables (age and Ki67 index) were compared across HER2 expression groups using the Kruskal–Wallis test. Due to the retrospective analysis of this study, no formal sample size calculation was conducted, and all statistics must be interpreted as descriptive by nature.

## 3. Results

### 3.1. Patterns of HER2 Expression and Descriptive Analyses

Overall, 57 maleBC patients were identified, and for 54 patients, full histology data was available, as described in the Methods section. The mean age was 64.3 years (SE ± 1.73). Regarding histology subtypes, 50/54 patients (92.6%) had NST carcinoma, while two patients (3.7%) had lobular and one patient (1.8%) had medullary BC, respectively. Out of all patients, 56/57 (98.2%) had luminal BC. No case of triple-negative BC was observed; 5/52 (9.6%) of patients had tumors showing HER2-positivity (HER2 3+ or 2+/ISHpos), of which one was non-luminal. Reassessments of IHC stainings of all 24 tumors historically reported as “IHC 0” or “negative” were performed. Here, 79.2% (19/24) tumors had either HER2-low or -ultralow expression. In the whole cohort, rates of HER2-low and -ultralow expression were 75.0% (39/52) and 5.8% (3/52), respectively (Table 1).

Three patients (5.3%) presented with primary metastatic disease. Genetic testing for hereditary BC was not performed or reported in 63.2% (36/57) of all patients, and germline *BRCA1/2* mutations were detected in 6 of 57 patients (10.5%).

**Figure 1:**
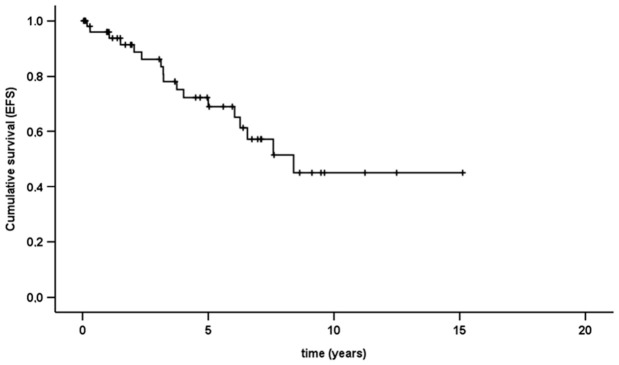
Event-free survival (EFS) over time (years) for the study population. *y*-axis shows percentage of total. Median EFS was 8.39 years (95% CI 7.36–11.35). Cross symbols indicate censoring.

The median follow-up was 7.0 years. Within the population of 54 patients with early-stage breast cancer, recurrences were registered in 13 patients (24.1%); 3-year and 5-year EFS as defined in the methods section was 86.9% and 71.0%, respectively (Figure 1). In the total population of 57 patients, including three cases of de novo metastatic disease, six deaths were counted (10.5%).

**Figure 2:**
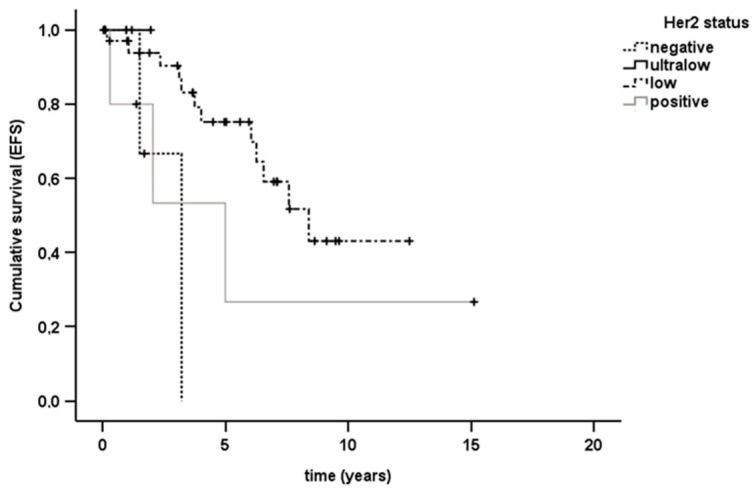
EFS over time by HER2 expression status. *y*-axis shows percentage of total. Cross symbols indicate censoring.

No statistically significant associations were observed between HER2 expression categories and grade, survival status, ER status, nodal status, or genetic variant status (all *p* > 0.05). Furthermore, no significant differences in age (*p* = 0.207) or Ki67 proliferation index (*p* = 0.897) were observed across HER2 expression groups (Table S1). Kaplan–Meier analysis showed no significant difference in EFS between HER2 expression (log-rank test, *p* = 0.133, Figure 2) or genetic variant groups (log-rank test, *p* = 0.691, Figure S1).

### 3.2. Locoregional Therapy

Surgery was performed in 54 (94.7%) of patients and all had radical mastectomy. Additional axillary dissection was performed in 37.0% (20/54 patients, Table 2). Upon surgery, one patient presented with carcinoma in situ (DCIS) and one had DCIS with microinvasion. The median tumor size was 21 mm; the median Ki67 positivity was 30%. Nodal metastasis was found in 40.7% (22/54) of patients undergoing surgery. Postoperative bleeding was observed in 9 of 54 patients (16.7%) but all complications were mild in severity (Clavien-Dindo Grade 1). The vast majority (*n* = 50 of 54, 92.6%) of patients undergoing surgery presented with luminal disease and ductal carcinoma or no special type (NST). Two patients had the lobular subtype, and one patient had the medullary subtype (Table 3).

### 3.3. Systemic Therapy

Within the total population, 20 patients (35.1%) received chemotherapy and 50 patients (87.7%) went on to receive endocrine therapy (Table 4). In addition, 13 patients (22.8%) were given radiotherapy. Endocrine treatment was administered in 50 of 57 patients (87.7%) and 3 of 57 patients (5.3%) received ET in combination with a CDK4/6 inhibitor. Additionally, gonadotropin-releasing hormone (GnRH) analogs were given in 15.8% of patients.

## 4. Discussion

In this retrospective study of maleBC patients treated at a tertiary center, high rates of low and ultralow HER2 expression were found. Additionally, we observed high rates of estrogen-receptor expression and a low rate of invasive lobular disease. Despite predominantly luminal and early biology, five-year EFS was low at 71%. No statistically significant differences in survival or association with clinicopathological characteristics were found for HER2 expression categories.

Our findings are of particular clinical interest in an evolving landscape of HER2-targeted ADCs in BC. Recent trials in female BC [13,21] have demonstrated clinically meaningful activity of ADCs in tumors with low and ultralow HER2 expression, thereby expanding the therapeutic relevance of refined HER2 categorization beyond the traditional HER2-positive/negative dichotomy.

To date, it remains unclear whether these findings can be extrapolated to maleBC. As DESTINY-Breast06 allowed for inclusion but failed to recruit maleBC patients into the ultralow treatment cohort [21], we hypothesized that the number of male patients exhibiting HER2-ultralow expression in their tumors must be low. Indeed, we found that only a relatively small portion—5.8%—of maleBC patients treated at our tertiary cancer within the last twenty years exhibited HER2-ultralow expression. Looking at published data on the prevalence of HER2-low expression in maleBC, this might be attributed to numerically higher numbers of HER2-low expression in men when compared to women. Of note, Ignatov et al. published the biggest maleBC dataset (*n* = 659 patients) to date describing HER2-low expression and found rates of 76% [15]. One might argue that other research has shown lower levels of HER2-low expression in maleBC; however, these cohorts comprise significantly less patients [16,17]. The prevalence of Her-low expression postulated by Ignatov et al. correlates with the finding of HER2-low expression in three quarters of maleBC included in our analysis and exceeds the numbers expected from female BC [22,23]. As maleBC is almost always HR-positive (98.3% in this analysis), previously described higher rates of HER2-low expression in HR-positive disease compared to triple-negative disease and an underlying HR/HER2 crosstalk may be causal for this observation [24,25,26].

Of note, HER2 expression—especially when low—may be dynamic [20] and differences in expression between low and ultralow levels may not be clinically relevant when treating with T-DXd, as data from the phase II trial DAISY suggests [27]. In our analysis, we were not able to provide data about changes in HER2 expression over time, which is why further and larger analyses examining this effect are dearly needed.

The strengths of our analysis lie in the long follow-up and almost complete clinicopathological data of patients included. The fact that, upon reassessment of historically “HER2 0/negative” IHC scores for this analysis, 79.2% (19/24) of cases were found to be either HER2-low or -ultralow calls for further analyses of larger previously published cohorts. Looking at our results, we argue that reassessments of HER2 expression status in clinical routine using archival tissue and/or repeated biopsies are key when evaluating maleBC patients for potential HER2-directed ADC therapy.

The descriptive analyses performed within our patient cohort show results in line with previously published data from maleBC cohorts. These include high rates of nodal involvement and comparably more aggressive disease as seen in our data [28]. Besides the lack of awareness and misconceptions about the disease, this may be attributed to the fact that men seek medical consultation from doctors, including general practitioners and medical oncologists, less often compared to women [29]. Prognosis in our study population, with 3- and 5-year EFS rates of 86.9% and 71.0%, was less favorable than rates seen in predominantly female and high-risk HR-pos trial populations of stage II/III BC, such as the control arms of monarchE and NATALEE [30,31]. Data from germline *BRCA* testing, which found six carriers of pathogenic BRCA1/2 mutations, was incomplete for our cohort due to routine genetic assessments not being performed until recently and due to legal regulations preventing precise documentation. Results should therefore be interpreted with caution.

The treatment data of our cohort closely reflects previously described characteristics of maleBC, as all patients undergoing surgery for localized disease underwent mastectomy. Therefore, use of post-mastectomy radiotherapy was low and restricted to patients with nodal disease. The relatively high rates of postoperative bleeding observed need to be validated in larger cohorts. High rates of chemotherapy use in our retrospective analysis replicate more advanced/node-positive disease in maleBC compared to its female counterpart.

Lastly, our study was limited by its retrospective design and small sample size, particularly within some HER2 expression subgroups, which may have reduced power and hindered statistical analyses besides descriptive methods. Therefore, analyses were primarily descriptive and exploratory in nature, limiting the ability to draw definitive conclusions regarding clinicopathological or prognostic associations. The lack of blinded reassessment of HER2 IHC scores represents a limitation of this study. However, strict blinding was not feasible in this real-world cohort, as the participating pathologists were familiar with many of the cases and had been involved in the original historical HER2 assessment. A further limitation is that HER2 reassessment was performed primarily in tumors historically classified as HER2 “0” or “negative”, reflecting the study aim to identify HER2-low and HER2-ultralow expression according to contemporary criteria. This selective reassessment may have introduced an upward bias in the observed prevalence of HER2-low disease, and the reported frequencies should therefore be interpreted with caution. Additionally, inclusion of all male patients, including those with metastatic and early disease, two cases of DCIS, available and missing HER2 information and their combined analysis, may have increased heterogeneity within our cohort. We argue, however, that from an ethical standpoint, cases of an understudied/orphan disease should always be reported and that statistical methods used were used appropriately and described meticulously.

## 5. Conclusions

We present single-center data of maleBC patients observed within two decades and describe high frequencies of HER2-ultralow and -low expression upon reassessment of archival IHC slides. This is of high clinical interest as potential applicability of ADC-based treatment strategies in maleBC currently remains extrapolated from studies conducted in female cohorts. Our findings highlight clinicopathologic, demographic as well as follow-up data and raise awareness for this rare patient population. Prospective, multicenter trials are needed to verify our findings using larger study cohorts in maleBC.

## Figures and Tables

**Table 1:** Patterns in HER2 expression according to IHC. HER2-positive—HER2 2+/ISHpos or HER2 3+.

*n* = 52	*n* (% of Total)
HER2-positive	5 (9.6%)
HER2-low	39 (75.0%)
HER2-ultralow	3 (5.8%)
HER2 0	5 (9.6%)

**Table 2:** Surgical data including type of surgery and complication rates. mins—minutes.

*n* = 54	*n* (% of Total)
Mastectomy	54 (100.0%)
Axillary dissection	20 (37.0%)
Local complication excl. bleeding	5 (9.3%)
Postoperative bleeding	9 (16.7%)
Median duration of surgery (mins)	70

**Table 3:** Clinicopathological characteristics of maleBC patients undergoing surgery for early disease.

*n* = 54	*n* (% of Total)
Tis	2 (3.7%)
T1	22 (40.7%)
T2	21 (38.9%)
T3/4	9 (16.7%)
N0	24 (44.4%)
N1-3	22 (40.7%)
Unknown	10 (18.5%)
lumA	8 (14.8%)
lumB	42 (77.8%)
HER2-positive	4 (7.4%)
TNBC	0 (0.0%)
NST	50 (92.6%)
other histological type	4 (74%)

**Table 4:** Treatments administered and BRCA mutations found in the total population of maleBC patients. CHT—chemotherapy; RT—radiotherapy; ET—endocrine treatment; Tam—tamoxifen; CDK4/6i—cyclin-dependent kinase 4/6 inhibitor; GnRH—gonadotropin-releasing hormone; gBRCAmut—germline BRCA1/2 mutation.

*n* = 57	*n* (% of Total)
Chemotherapy (CHT) use	20 (35.1%)
No CHT	32 (56.1%)
Unknown	5 (8.8%)
Neoadj. CHT	7 (12.3%)
Radiotherapy (RT)	13 (22.8%)
No RT	39 (68.4%)
Unknown	5 (8.8%)
Endocrine treatment (ET)	50 (87.7%)
No ET	3 (5.3%)
Unknown	4 (7.0%)
Tamoxifen	45 (78.9%)
Tam + CDK4/6i	3 (5.3%)
Other	1 (1.8%)
GnRH addition	9 (15.8%)
gBRCA1/2mut	6 (10.5%)

## Data Availability

The data presented in this study are available on request from the corresponding author and restricted due to ethical and legal reasons.

## Supplementary Materials

The following supporting information can be downloaded at: https://www.mdpi.com/article/10.3390/life16060947/s1, Figure S1: Association between genetic variation and event-free survival (EFS) over time.; Table S1: Association between HER2 expression categories and clinicopathological characteristics.
