# Supplementary material for: Characterization of HER2 Expression Levels, Including HER2-Ultralow, in a Retrospective Male Breast Cancer Cohort
Source: Life (Basel). 2026 Jun 3;16(6):947. doi: 10.3390/life16060947 (PMC13301673; doi:10.3390/life16060947)
Supplement: Supplementary file 1 [file life-16-00947-s001.zip › life-4317511-supplementary.pdf]

| Characteristic | HER2-negative (n=5) | HER2-ultralow (n=3) | HER2-low (n=39) | HER2-positive (n=5) | p-value |
|---|---|---|---|---|---|
| **Grade** | | | | | **0.530** |
| G1 | 0 (0%) | 0 (0%) | 3 (7.7%) | 0 (0%) | |
| G2 | 2 (40%) | 2 (66.7%) | 17 (43.6%) | 0 (0%) | |
| G3 | 3 (60%) | 1 (33.3%) | 18 (46.2%) | 5 (100%) | |
| **ER positive, n (%)** | 5 (100%) | 3 (100%) | 39 (100%) | 4 (80%) | **0.250** |
| **Nodal status** | | | | | **0.616** |
| Positive | 3 (60%) | 0 (0%) | 16 (41.0%) | 1 (20%) | |
| Negative | 2 (40%) | 2 (66.7%) | 17 (43.6%) | 3 (60%) | |
| Unknown | 0 (0%) | 1 (33.3%) | 6 (15.4%) | 1 (20%) | |
| **OS event, n (%)** | 1 (20%) | 0 (0%) | 5 (12.8%) | 0 (0%) | **0.840** |
| **Genetic variant** | | | | | **0.506** |
| Yes | 1 (20%) | 1 (33.3%) | 4 (10.3%) | 0 (0%) | |
| No | 0 (0%) | 1 (33.3%) | 11 (28.2%) | 1 (20%) | |
| Unknown | 4 (80%) | 1 (33.3%) | 24 (61.5%) | 4 (80%) | |
| **Age, median (Q1–Q3)** | 76 (60.5–79.5) | 81 (71–NA) | 61 (55.8–72.5) | 66 (55–74.5) | **0.207** |
| **Ki67, median (Q1–Q3)** | 40 (25–50) | 30 (30–NA) | 32.5 (20–50) | 30 (24.5–55) | **0.897** |

**Table S1 - Association between HER2 expression categories and clinicopathological characteristics**.

Categorical variables were analyzed using the Fisher–Freeman–Halton exact test. Continuous variables were analyzed using the Kruskal–Wallis test. Q1–Q3: interquartile range.

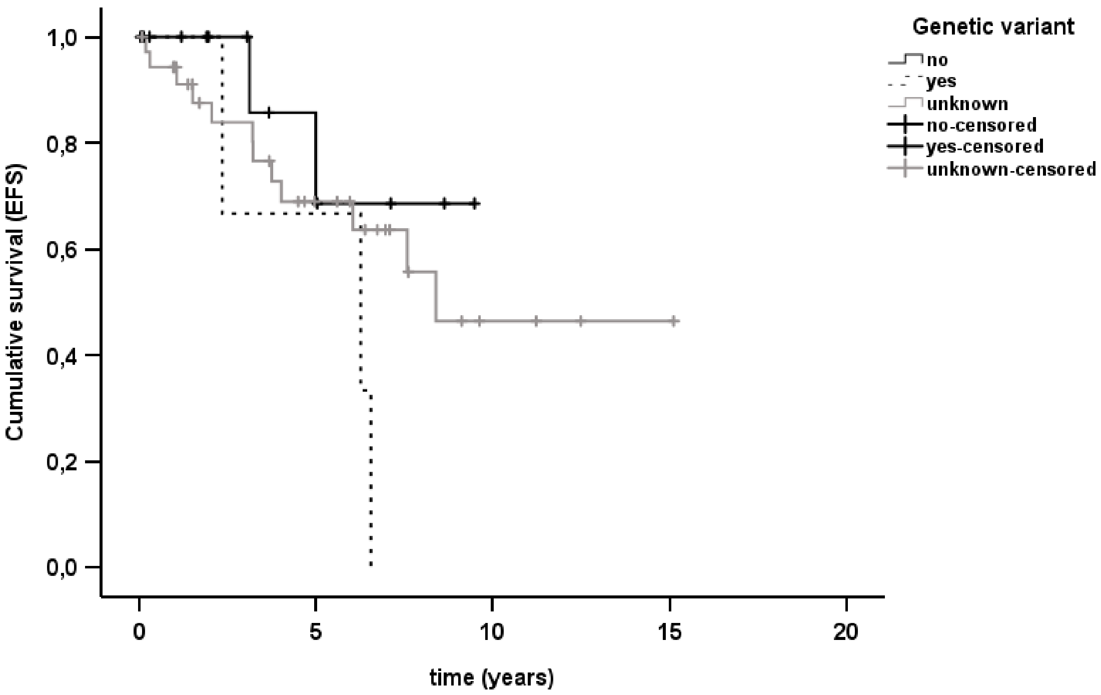


**Figure S1 - Association between genetic variation and event-free survival (EFS) over time.**

Y-axis shows percentage of total. No statistically significant differences in survival by genetic variant status were found within the study cohort (log-rank test, $p = 0.691$).
